# Risk of Budd-Chiari Syndrome Associated with Factor V Leiden and G20210A Prothrombin Mutation: A Meta-Analysis

**DOI:** 10.1371/journal.pone.0095719

**Published:** 2014-04-22

**Authors:** Peijin Zhang, Jing Zhang, Guixiang Sun, Xiuyin Gao, Hui Wang, Wenjun Yan, Hao Xu, Maoheng Zu, He Ma, Wei Wang, Zhaojun Lu

**Affiliations:** 1 Department of Public Health, Xuzhou Medical College, Xuzhou, Jiangsu, China; 2 Department of Interventional Radiology, Affiliated Hospital of Xuzhou Medical College, Xuzhou, Jiangsu, China; University of Modena & Reggio Emilia, Italy

## Abstract

**Background:**

Various studies have demonstrated that factor V Leiden (FVL) and G20210A prothrombin mutation contribute to the risk of Budd-Chiari syndrome (BCS), while other studies provided conflicting findings. In order to derive more precise estimations of the relationships, a meta-analysis was performed.

**Methods:**

Eligible articles were identified through search of databases including Pubmed, Chinese Biomedical Database (CBM, Chinese), and Chinese National Knowledge Infrastructure (CNKI, Chinese). Odd ratios (ORs) with 95% confidence intervals (CIs) were calculated using random- or fixed- model.

**Results:**

Finally, twelve studies were included for FVL and nine studies were included for G20210A prothrombin mutation. With respect to FVL, significantly increased BCS risk was found in the overall population (OR = 6.29, 95%CI = 4.23–9.36). Subgroup analyses suggested that FVL was associated with an increased risk of BCS in the population with high background mutation prevalence (>1% in the normal population). No significant association was found between BCS and G20210A prothrombin mutation (OR = 1.78, 95%CI = 0.77–4.11).

**Conclusion:**

The presence of FVL should be evaluated in patients with BCS. Conversely, G20210A prothrombin mutation is not significantly associated with risk of BCS. Large-scale well designed studies are necessary to be conducted to further confirm or refute the observed association.

## Introduction

Splanchnic vein thrombosis (SVT) encompasses hepatic vein thrombosis (Budd–Chiari syndrome, BCS), portal vein thrombosis (PVT), and mesenteric vein thrombosis. BCS is a rare but clinically challenging disorder defined as obstruction of hepatic venous outflow anywhere from the small hepatic veins to the suprahepatic inferior vena cava [1]. The incidence of BCS appears to vary according to the different study populations. For example, studies from France and Spain have shown incidence rates of 0.2 [2] and 0.41 per million inhabitants per year [3], respectively. However, BCS is the leading cause of hospitalization due to liver disease in developing countries such as Nepal and India [4], [5]. In China, BCS has a considerably high incidence rate, especially in Shandong, Henan, Anhui provinces, and north part of Jiangsu province [6], [7]. The pathogenesis of BCS is still not fully understood. Some factors such as myeloproliferative neoplasm, oral thrombotic contraceptives, infection, chronic inflammatory diseases, pregnancy, puerperium, poor nutrition are considered to be risk factors for BCS [8]–[10]. In the past decades, several inherited factors causing a hypercoagulable state have been studied in patients with venous thromboembolism (VTE). Resistance to activated protein C (APC) which was the most common cause of inherited thrombophilia was discovered in 1993 [11]. One year later, FVL was recognized as the frequent cause of this resistance [12]. Subsequently, a mutation in the prothrombin regulatory sequence was found to be another common prothrombotic factor in 1996 [13]. Several meta-analyses have confirmed these thrombophilic abnormalities are associated with an increased risk of VTE [14]–[16].

To date, numerous observational studies have reported the prevalence of FVL and G20210A prothrombin mutation in patients with BCS. But the prevalence of these mutations is widely varied in different studies. For example, the prevalence of FVL was found to range between 6.8–31.8% in BCS cases [17]–[20], while it was found to be zero in other studies [21]–[24]. The phenomenon is probably because each study uses its own eligibility criteria and sample sizes are small, and no quantitative syntheses of studies have been performed. Therefore, we conducted a meta-analysis of observational studies to obtain the most convinced estimates of the prevalences of FVL and G20210A prothrombin mutation and to evaluate the risk of BCS associated with these two inherited mutations.

## Methods

A protocol was prospectively performed, detailing the study objectives, predefined criteria for study selection and methods of statistical analysis.

### Search Strategy

A comprehensive search strategy was conducted towards the electronic databases including PubMed, Chinese Biomedical Database (CBM, Chinese) and Chinese National Knowledge Infrastructure (CNKI, Chinese) for relevant reports published before May 20, 2013. Searched items were presented in the File S1. Although no language restricts were applied initially, our final analysis only permitted the review of articles published in English and Chinese. Additional studies were identified by a hand search of the references of original studies; review articles were also examined to find additional eligible studies.

### Selection Criteria

We reviewed abstracts of all citations and retrieved studies. The following criteria were used to evaluate published studies: (1) evaluating the association between the two mutations and BCS; (2) case-control design; (3) the articles must offer the sample size, distribution of alleles or other information for estimating the odds ratio (ORs) and 95% confidence interval (CIs); (4) diagnosis of BCS was objectively confirmed (patients with BCS diagnosed with Doppler ultrasonography, computed tomography, magnetic resonance imaging, venography or patients who were diagnosed during laparotomy or abdominal surgery were considered eligible); and (5) the control groups were healthy subjects or patients without a history of thromboembolic disease or genetic relationship with the patients. Participants could be of any age. Studies were excluded if one of the following existed: (1) not a case–control study, (2) studies that contained duplicate data, (3) no usable data reported, and (4) case reports, reviews, comments or animal studies.

### Data Extraction

Two investigators (Zhang P, Wang H) independently extracted the following data: i) study characteristics (year of publication, design and study center); ii) patients and controls characteristics (number of subjects studied, mean age, variation in age, gender and race); iii) prevalence of FVL and G20210A prothrombin mutation in cases and in controls. In cases of disagreements between the two reviewers, a consensus was achieved by discussion among all of the reviewers.

### Study Validity Assessment

The quality of the articles that were eligible for inclusion in this review was assessed using an adapted 10-pointed Newcastle-Ottawa Scale (NOS) [25]. The quality of each study was assessed and awarded stars for indicators of quality by two authors (Zhang J, Wang H). Discrepancies between two authors were dealt with by a joint re-evaluation of the original article.

### Statistical Analysis

The strength of association between the two mutations and BCS risk was estimated by calculating summary crude odds ratios (ORs) and corresponding 95% confidence intervals (CIs). The Q test and I^2^ statistics were used to assess the statistical heterogeneity among studies [26], [27]. If the result of the Q test was *p*<0.1 or I^2^>50%, indicating the presence of heterogeneity, a random-effects model (DerSimonian-Laird method) was used to estimate the summary ORs [28]; otherwise, the fixed-effects model (Mantel-Haenszel method) was used [29]. Subgroup analyses were conducted based on the background mutation prevalence of thrombophilic abnormalities in the normal population. The potential small-study bias was tested using the Egger regression test asymmetry [30] and the Begg’s test for funnel plot, which was based on Kendall’s tau [31]. Sensitivity analysis was performed by omitting one study at a time to assess the influence of individual studies on our meta-analysis. All statistical tests were two-sided and *P*-values <0.05 were considered statistically significant. All analyses above were conducted using the STATA version 12.0 software (Stata Corp, College Station, TX).

The proportion of BCS cases in the population that could be attributed to FVL or G20210A prothrombin mutation (population-attributable risk [PAR] was estimated from the fixed effects model and the prevalence of exposure was estimated as the genotype frequency among control subjects using the following formula:
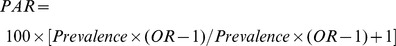



## Results

### Study Identification and Selection

**Figure 1:**
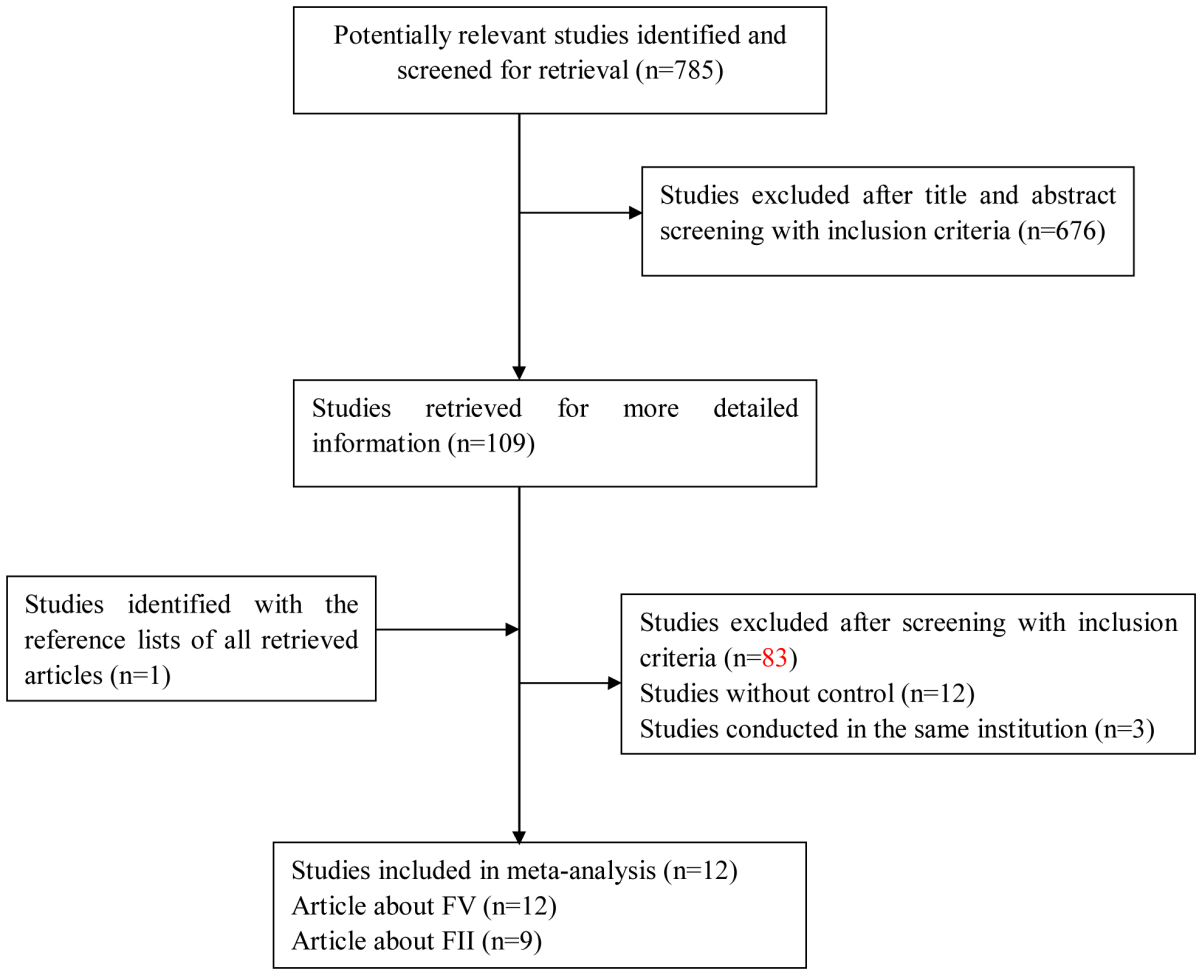
Flow chart of the selection process for including article.

The PRISMA checklist was presented as Checklist S1. The literature strategy yielded 785 results, including 735 in the PubMed database, 41 in the CNKI, and 9 in the CBM. Of these, we excluded 676 articles after title and abstract screening using the prespecified inclusion and exclusion criteria, and 109 articles were retrieved for more detailed assessment (Fig. 1). Finally, fourteen of them met our eligibility criteria [23], [24], [32]–[43]. Additionally, one study was identified by manually reviewing the references of the eligible articles retrieved [44].

Among the fourteen studies three were reported in the same institution [24], [34], [37], and we at last chose the case series with more complete data and more extensive interval of enrollment to calculate the pooled OR [24]. The study [38] was further excluded from our research in the same way. Altogether, twelve studies [23], [24], [32], [33], [35], [36], [39]–[44] which contained 516 cases and 1342 controls were included in the final analysis (Table S1). All included studies were published in English, except three [23], [24], [40] in Chinese.

### Study Quality

The NOS for assessing the quality of included studies is shown in Table S2, with scores ranging from 4–8. Eight of the case-control studies were awarded the maximum of four stars based on the selection of the study population. Five studies were awarded one star in the score for comparability, when mentioned adjusted only for age and sex. All the case-control studies were awarded the maximum of three stars in exposure assessment.

### Factor V Leiden (FVL)

**Figure 2:**
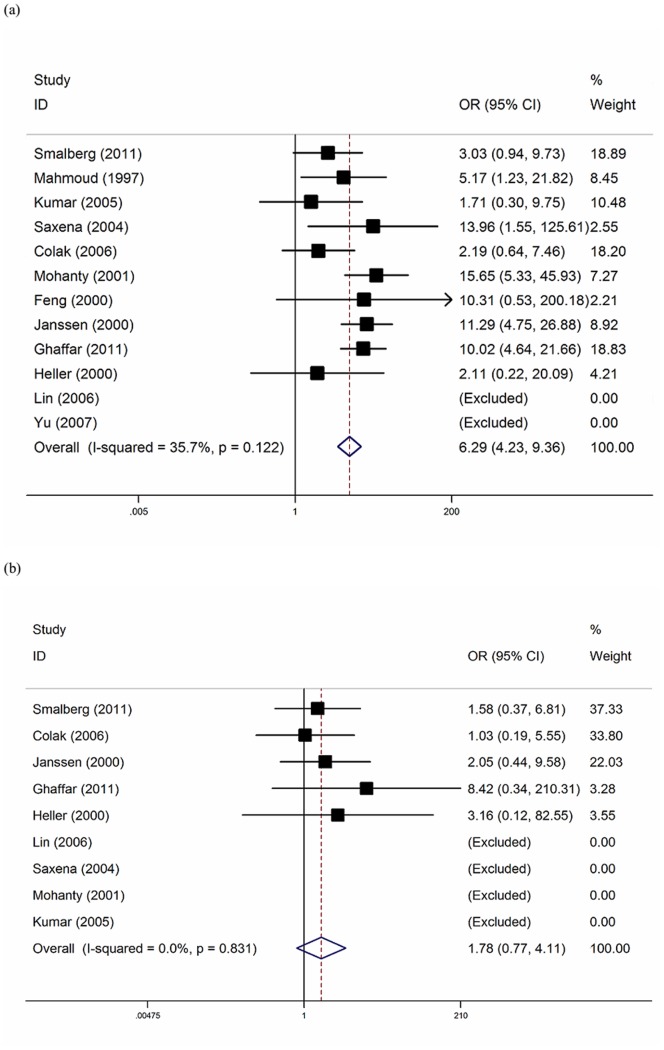
Forest plot of the association between BCS and FVL (a), and G20210A prothrombin mutation (b).

**Figure 3:**
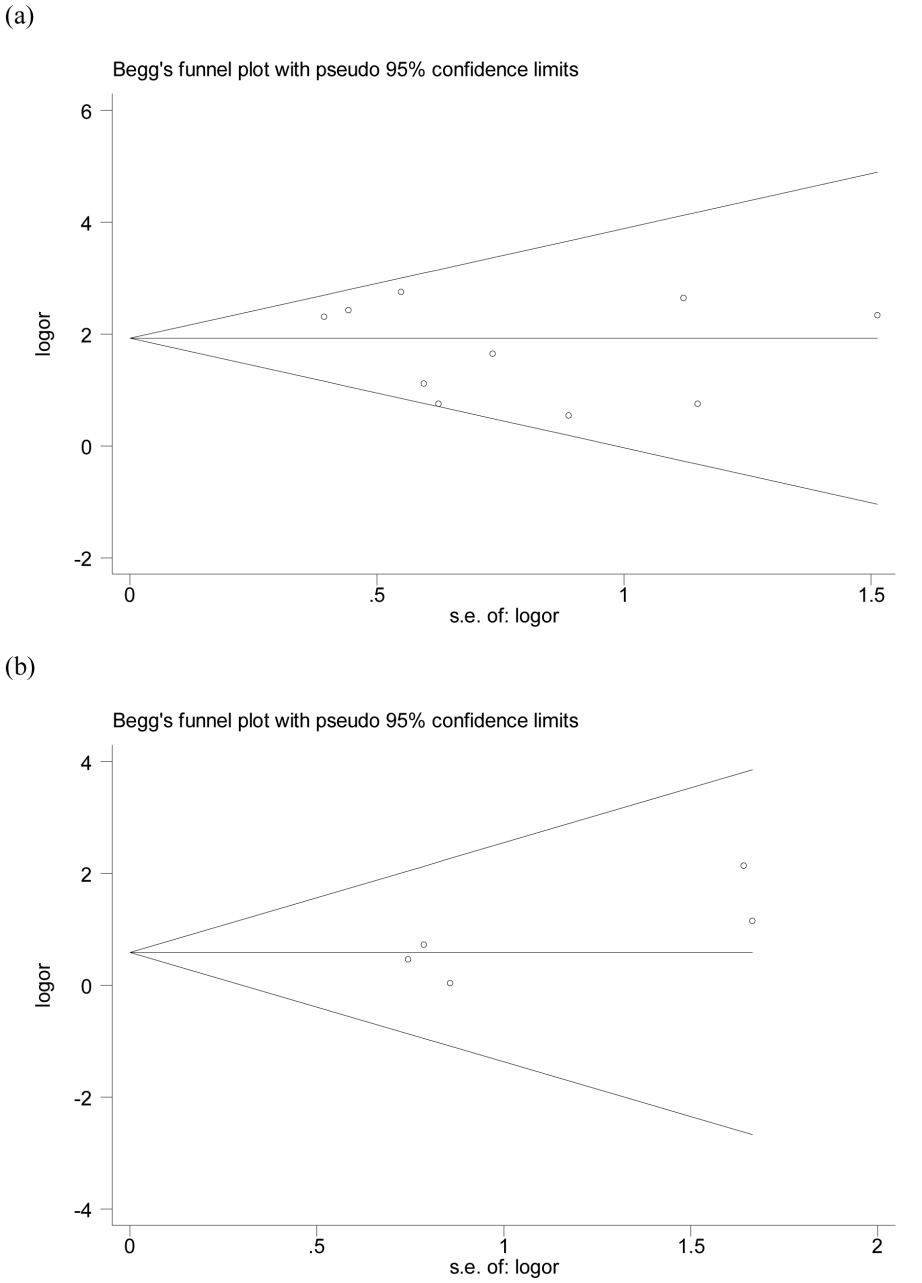
Begg’s funnel plot: publication bias for the studies on FVL (a), and on G20210A prothrombin mutation (b).

In the overall analysis, the FVL mutation was significantly associated with elevated risk of BCS with a pooled OR of 6.29 (95%CI = 4.23–9.36) (Fig. 2a). There was no significant heterogeneity between the studies (I^2^ = 35.7%). The funnel plot appeared symmetric, suggesting no distinct small-study bias among the studies existed (Egger’s *p* = 0.26) (Fig. 3a). The estimated attributable risk of BCS conferred by FVL mutation was 18.35% in this pooled cohort.

Nine studies [32], [33], [35], [36], [39], [41]–[44] provided data on the prevalence of FVL in the population with high background mutation (>1% in the normal population [45], [46]). In this population, FVL was associated with an increased risk of BCS with a pooled OR of 6.20 (95%CI = 4.15–9.26). The heterogeneity between the studies was not significant (I^2^ = 42.7%). There was no distinct small-study bias among the studies (Egger’s *p* = 0.16). This was supported by the symmetric funnel plot (figure not shown) of precision of the log [OR] in relation to its standard errs. The estimated attributable risk was 19.41%.

### G20210A Prothrombin Mutation

Nine studies evaluated the role of G20210A prothrombin mutation in the risk of BCS [24], [32], [33], [35], [36], [39], [41], [42], [44]. In this population the presence of G20210A prothrombin mutation did not result to be statistically significant associated with an increased risk of BCS with a pooled OR of 1.78 (95%CI = 0.77–4.11) (Fig. 2b). No G20210A prothrombin mutations were found in four studies [24], [35], [36], [39]. There was no significant heterogeneity between the studies (I^2^ = 0.0%). The funnel plot appeared symmetric, suggesting no distinct small-study bias among the studies existed (Egger’s *p = *0.15) (Fig. 3b). The estimated attributable risk of BCS conferred by G20210A prothrombin mutation was 1.11% in this pooled cohort.

### Sensitivity Analyses

**Figure 4:**
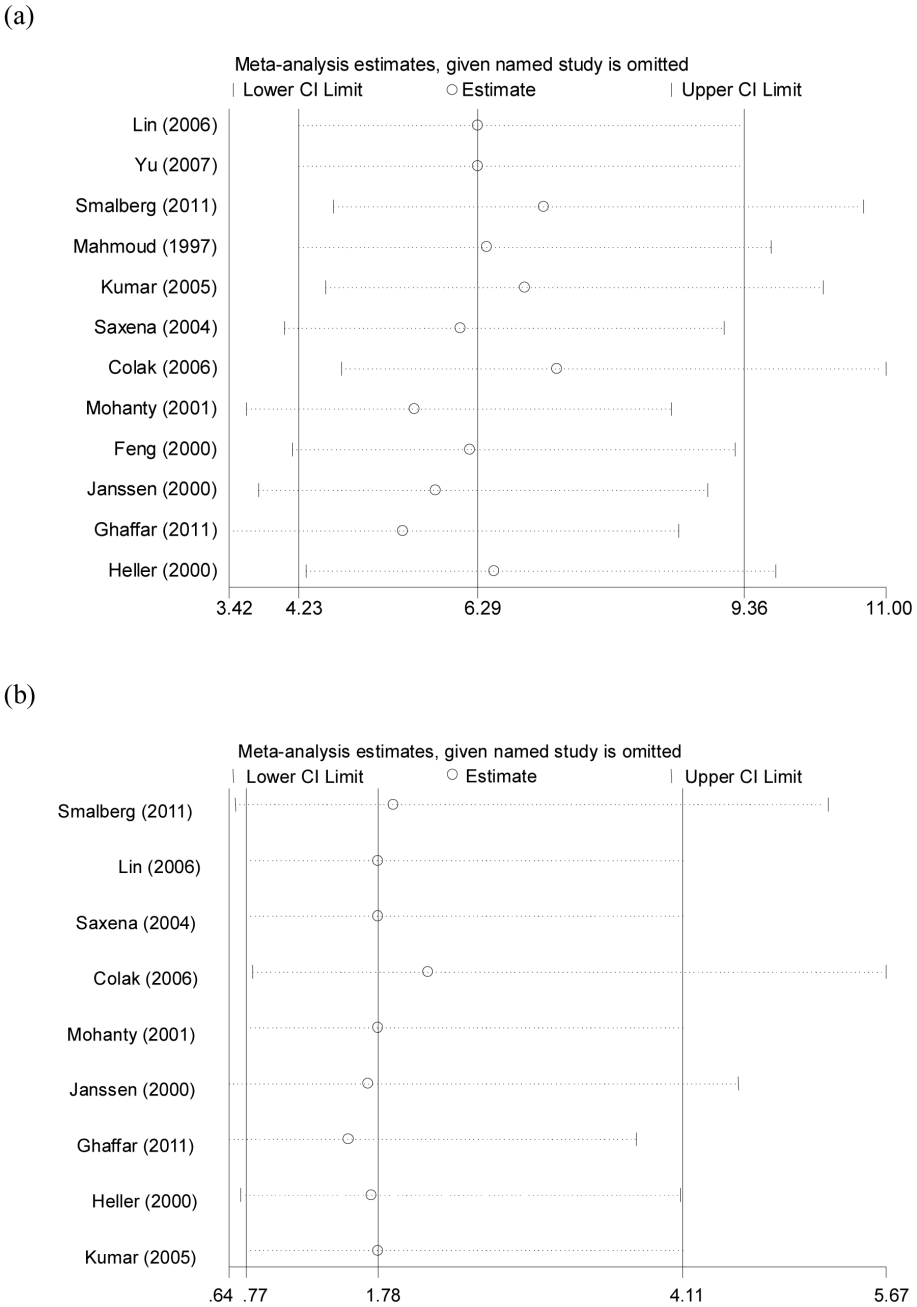
Sensitivity analysis of the association between BCS and FVL (a), and G20210A prothrombin mutation (b).

In order to assess the stability of the results of the meta-analysis, we conducted a sensitivity analysis through sequentially excluded individual studies. Statistically similar results were obtained after sequentially excluding each study, confirming the stability of the meta-analysis (Fig. 4). So, results of the sensitivity analyses suggest that the data in our meta-analysis are relatively stable and credible.

## Discussion

Inherited deficiency of antithrombin (AT), protein C (PC) and its co-factor, protein S (PS) were the major known genetic defects detected for VTE. The defect in the anticoagulation response to APC had been detected as a new mechanism for thrombophilia, which was subsequently linked to a single point mutation on the factor V gene, resulting in Arg506-Gln substitution in the APC cleavage site [12]. FVL as a causative mutation for APC and consequently to thrombosis has been one of the major advances for the understanding of the pathogenesis of thrombotic disorders including BCS.

On the other hand, thrombin (clotting factor II) usually produced in the liver in an inactive form called prothrombin, is a robust enzyme that plays a major role in the coagulation system by activating many clotting factors and other elements of the coagulation system like the blood platelets. Poort et al [13] performed an extensive DNA sequencing on the prothrombin gene (on chromosome 11) for patients with unexplained VTE and found a single missense mutation (G to A) at nucleotide position 20210, which was present in the 3′ untranslated region of the prothrombin gene. Bucciarelli et al [47] firstly reported the BCS patient with heterozygous G20210A prothrombin mutation in 1998. Since then, some scholars tried to explore the relationship between BCS and prothrombin G20210A mutation. A previously published meta-analysis [48] involving more than 3000 patients with PVT showed a strong association with G20210A prothrombin mutation (OR = 4.48, 95%CI = 3.1–6.48) and FVL (OR = 1.9, 95%CI = 1.25–2.9), respectively. More recently, a published meta-analysis assessed the prevalence of AT, PC and PS deficiencies in PVT and BCS [49]. They concluded inherited AT, PC and PS deficiencies were rare in BCS and PVT, and the inherited deficiencies increased the risk of PVT. However, we should notice that there was only one case-control study about BCS.

Our system review of the literature and meta-analysis with twelve studies indicated that FVL was more common in patients with BCS than control subjects, suggesting a strong association between BCS and FVL. Interestingly, the frequency of FVL depended on the geographical location and the ethnic background of the population. Eroglu [50] proposed that the frequency of FVL was high in the Middle East, Southern Europe, Mediterranean region and Indian population, while it was low in other populations like Chinese, Japanese, Korean [45], [46], [51]. Therefore, we performed a subgroup analysis by the proportion of frequency. The nine studies in English enrolled participants all from the region with higher frequency of FVL. The pooled OR was very close to the whole OR, but the PAR was greater, suggesting routine screening for FVL was potentially warranted in clinical practice in these regions. On the other hand, no mutations were found in the two [23], [24] of another three studies in Chinese. Several recent observational studies did not find FVL in BCS cases [21], [22], [52] and healthy population in China [53], [54] which was consistent with the opinion proposed by Rees [51] and Jadaon [46]. Surprisingly, the remained study [40] in Chinese indicated FVL was tested in familial BCS patients. Four heterozygous FVL were found in the whole six familial BCS cases that were from northeast region China. However, this result could not be reproduced by another familial study [24]. No mutations were found in four familial BCS cases from Henan province in Yellow River valley which was considered as areas with high BCS prevalence in China [55]. We speculate this difference may be caused due to environmental factors, and genetic changes can not play pathogenic roles individually in determining the risk of familial BCS. Therefore, further studies are needed to confirm the concise relation between FVL and familiar BCS patients in China.

Conversely, G20210A prothrombin mutation did not result to be statistically significant associated with an elevated risk of patients with BCS, suggesting no association between BCS and G20210A prothrombin mutation. G20210A prothrombin mutation was not found in four of nine studies. It was noteworthy that the four studies all came from Asian countries. On the other hand, the mutation is present in 0.7–4% of the general population and up to 8% of deep vein thrombosis (DVT) patients [56] in Western countries, while in India and many other Asian countries, it has not been detected [57]–[59]. It maybe suggested that screening for this mutation was generally not indicated in Asian populations. Result of this analysis may be due to the small number of included patients and the variation of geographic distribution. Therefore, our results should be interpreted with caution. In addition, further case-control studies should be actively performed to explore the relationship between G20210A prothrombin mutation and BCS in larger numbers. Together, results of this meta-analysis suggest the presence of FVL should be evaluated in patients with BCS in the Middle East, Southern Europe, Mediterranean region and Indian population. On the other hand, the role of G20210A prothrombin mutation in patients with BCS is less clear.

As with any meta-analysis there are a number of limitations which need to be considered. Firstly, our meta-analysis was restricted to case-control studies, and the application of formal meta-analytic methods to observational studies is controversial, since bias implicit in the study design may distort the strength of association within the data. We therefore chose only studies in which the diagnosis of BCS was objectively confirmed to reduce the potential bias. Secondly, the majority of the studies were conducted in Caucasian population, so there might be a bias for the entire population and our results should be extrapolated with caution. Thirdly, in a meta-analysis of published studies, it was possible that an observed association might suffer from publication bias because studies with null results tend not to be published. However, no significant publication bias was detected in this meta-analysis. Finally, our results should be extrapolated with caution in BCS patients with G20210A prothrombin mutation, since G20210A prothrombin mutation was detected in small samples in our meta-analysis. Further case-control studies should be performed to explore the risk between G20210A prothrombin mutation and BCS.

Despite the limitations above, our meta-analysis also had several advantages: First, we gathered all the available studies on the association between genetic thrombophilic mutations and the risk of BCS. For the first time, a meta-analysis evaluated factor V Leiden and prothrombin mutation specifically in the context of BCS. Second, our meta-analysis of the association between inherited thrombophilic abnormalities and BCS risk is statistically more powerful than any other single study. Third, the quality of case–control studies included in the meta-analysis met our inclusion criteria and was satisfactory, and the sensitivity analysis indicated that the results of our meta-analysis were stable and convincing.

In summary, the meta-analysis indicates that, BCS is associated with the presence of FVL in the Middle East, Southern Europe, Mediterranean region and Indian population and routine screening of FVL appears to be indicated in patients with BCS. Conversely, G20210A prothrombin mutation is not significantly associated with risk of BCS. Large-scale well designed studies are necessary to be conducted to further confirm or refute the observed association.

## Supporting Information

Table S1 **Baseline characteristics of the studies included in the meta-analysis.**
(DOC)

Table S2 **Newcastle-Ottawa Scale (NOS) for assessing quality of non-randomized studies.**
(DOC)

File S1 **Pubmed search strategy.**
(DOC)

Checklist S1 **Reporting items for the meta-analyses.**
(DOC)
