# Supplementary material for: Risk of Budd-Chiari Syndrome Associated with Factor V Leiden and G20210A Prothrombin Mutation: A Meta-Analysis
Source: PLoS One. 2014 Apr 22;9(4):e95719. doi: 10.1371/journal.pone.0095719 (PMC3995749; doi:10.1371/journal.pone.0095719)
Supplement: Table S1 — Baseline characteristics of the studies included in the meta-analysis. (DOC) [file pone.0095719.s001.doc]

**Table S1**. Baseline characteristics of the studies included in the meta-analysis

| Author | Study | Presence of thrombo- | | | Cases' mean | Cases description | Controls' | Controls description |
| --- | --- | --- | --- | --- | --- | --- | --- | --- |
| (year) | design | philic abnormalities | | | or median |  | mean or |  |
|  |  | RF | Cases | Controls | age years |  | median age |  |
|  |  |  | (n/N) | (n/N) | (range) |  | years(range) |  |
| Lin(2006) | Case- | FV | 0/49 | 0/70 | 32(9-59) | Patients with BCS | 29(19-48) | Healthy individuals |
|  | control | FII | 0/49 | 0/70 |  |  |  |  |
| Yu(2007) | Case- | FV | 0/26 | 0/10 | NA | Confirmed by surgery | NA | NA |
|  | control |  |  |  |  |  |  |  |
| Feng(2000) | Case- | FV | 4/31 | 0/31 | 32.3 | Confirmed by IVC | NA | Healthy subjects |
|  | control |  |  |  |  | radiography |  |  |
| Smalberg | Case- | FV | 12/107 | 4/100 | 38.1(28-51) | confirmed by | 26.8(27-50) | Healthy, unrelated, |
| （2011） | control | FII | 5/107 | 3/100 |  | radiographic |  | population-based |
|  |  |  |  |  |  | imaging |  | controls |
|  |  |  |  |  |  | (US ,CT, MRI ,or |  |  |
|  |  |  |  |  |  | venography) |  |  |
| Mahmoud(1997) | Case- | FV | 7/30 | 3/54 | 33(16-64) | Patients with BCS | 43(17-69) | Patients with hepatic |
|  | control |  |  |  |  |  |  | disorders and no history |
|  |  |  |  |  |  |  |  | of thrombotic disease |
| Kumar(2005) | Case- | FV | 4/59 | 2/49 | 27(5-69) | Diagnosed by clinical | NA | Healthy adult subjects |
|  | control | FII | 0/59 | 0/49 |  | and radiological |  |  |
|  |  |  |  |  |  | criteria |  |  |
| Saxena (2004) | Case- | FV | 5/29 | 1/68 | NA | Confirmed by | NA | Healthy age- and |
|  | control | FII | 0/29 | 0/68 |  | appropriate radio graphic |  | sex-matched persons |
|  |  |  |  |  |  | imaging such as Doppler |  |  |
|  |  |  |  |  |  | US, CT, MRI ,or Venography |  |  |
| Colak(2006) | Case- | FV | 9/32 | 5/33 | (19-45) | Diagnosed by Doppler | (19-45) | Age-and sex-matched |
|  | control | FII | 3/32 | 3/33 |  | ultrasound and venography |  | healthy volunteers |
| Mohanty(2001) | Case- | FV | 14/52 | 5/223 | NA | The confirmation was done | NA | Normal age-matched |
|  | control | FII | 0/52 | 0/223 |  | by Doppler sonography, |  | healthy subjects who |
|  |  |  |  |  |  | MRI, CT and venography. |  | did not have any |
|  |  |  |  |  |  |  |  | history of thrombosis |
|  |  |  |  |  |  |  |  | or any medication |
| Janssen(2000) | Case- | FV | 11/43 | 14/474 | 40(19-60) | Confirmed by appropriate | 47(16-73) | Healthy individuals who |
|  | control | FII | 2/43 | 11/474 |  | radiographic |  | had no history of venous |
|  |  |  |  |  |  | abdominal imaging(i.e. |  | thromboembolism, age |
|  |  |  |  |  |  | Doppler US,CT,MRI |  | sex ethnic descent |
|  |  |  |  |  |  | venography or laparotomy) |  | of controls and the |
|  |  |  |  |  |  |  |  | patients were similar |
| Ghaffar | Case- | FV | 29/47 | 18/130 | NA | Confirmed via | NA | Healthy volunteers |
| (2011) | control | FII | 1/47 | 0/130 |  | appropriate abdominal |  | without a family history |
|  |  |  |  |  |  | radiographic imaging |  | of thrombosis |
|  |  |  |  |  |  | (diagnostic criteria) |  |  |
| Heller(2000) | Case- | FV | 1/10 | 5/100 | NA | Confirmed by standard | NA | Age- and sex-matched |
|  | control | FII | 0/10 | 1/100 |  | imaging methods |  | healthy subjects |
|  |  |  |  |  |  | (Duplex sonography, |  |  |
|  |  |  |  |  |  | CT, angiography, MRI) |  |  |

RF, risk factor; FV, factor V Leiden; FII, G20210A prothrombin mutation; BCS, Budd-Chiari syndrome; CT, computed tomography; MRI, magnetic resonance imaging; US, ultrasonography; NA, not available.
