# Supplementary material for: Risk of Budd-Chiari Syndrome Associated with Factor V Leiden and G20210A Prothrombin Mutation: A Meta-Analysis
Source: PLoS One. 2014 Apr 22;9(4):e95719. doi: 10.1371/journal.pone.0095719 (PMC3995749; doi:10.1371/journal.pone.0095719)
Supplement: Table S2 — Newcastle-Ottawa Scale (NOS) for assessing quality of non-randomized studies. (DOC) [file pone.0095719.s002.doc]

**Table S2.** Newcastle-Ottawa Scale (NOS) for assessing quality of non-randomized studies.

|  | Selection | | | |  | Comparability | |  | Exposure | | |  |
| --- | --- | --- | --- | --- | --- | --- | --- | --- | --- | --- | --- | --- |
|  |  |  |  |  |  | Comparability | Comparability |  |  |  |  |  |
|  |  |  |  |  |  | of selected | of selected |  |  | Same method |  |  |
|  | Is the |  |  |  |  | cases and | cases and |  |  | of |  |  |
| Non-RTC | case | Represent- | Selection | Definition |  | controls, | controls, |  |  | ascertainment | Non- | Total |
| studies: | definition | ativeness | of | of |  | main factor: | secondary |  | Ascertainment | for cases | response | quality |
| author (year) | adequate? | of cases | controls | controls |  | age/sex | factor |  | of exposure | and controls | rate | score |
| Lin(2006) | * | * | * | * |  |  |  |  | * | * | * | 7 |
| Yu(2007) | * |  |  |  |  |  |  |  | * | * | * | 4 |
| Feng(2000) | * |  | * | * |  |  |  |  | * | * | * | 6 |
| Smalberg（2011） | * | * | * | * |  |  |  |  | * | * | * | 7 |
| Mahmoud(1997) | * |  |  | * |  |  |  |  | * | * | * | 5 |
| Kumar(2005) | * |  | * | * |  |  |  |  | * | * | * | 6 |
| Saxena (2004) | * | * | * | * |  | * |  |  | * | * | * | 8 |
| Colak(2006) | * | * | * | * |  | * |  |  | * | * | * | 8 |
| Mohanty(2001) | * | * | * | * |  | * |  |  | * | * | * | 8 |
| Janssen(2000) | * | * | * | * |  | * |  |  | * | * | * | 8 |
| Ghaffar(2011) | * | * | * | * |  |  |  |  | * | * | * | 7 |
| Heller(2000) | * | * | * | * |  | * |  |  | * | * | * | 8 |

RTC, randomized controlled trial.
