# Supplementary material for: Risk of Budd-Chiari Syndrome Associated with Factor V Leiden and G20210A Prothrombin Mutation: A Meta-Analysis
Source: PLoS One. 2014 Apr 22;9(4):e95719. doi: 10.1371/journal.pone.0095719 (PMC3995749; doi:10.1371/journal.pone.0095719)
Supplement: File S1 — Pubmed search strategy. (DOC) [file pone.0095719.s003.doc]

**Pubmed search strategy:**

#1 Search: “hepatic” (Title/Abstract) or “inferior vena cava” (Title/Abstract) or “portal” (Title/Abstract) or “mesenteric” (Title/Abstract) or “mesentery” (Title/Abstract) or “spleen” (Title/Abstract) or “splenic” (Title/Abstract) or “Splanchnic” (Title/Abstract).

#2 Search: “thrombosis” (Title/Abstract) or “thrombus” (Title/Abstract) or “thrombi” (Title/Abstract) or “thrombin” (Title/Abstract) or “thrombosed” (Title/Abstract) or “thrombotic” (Title/Abstract) or “occlusive” (Title/Abstract) or “occlusion” (Title/Abstract) or “occluded” (Title/Abstract) or “obstruction” (Title/Abstract) or “obstructed” (Title/Abstract) or “stenosis” (Title/Abstract) or “stenotic” (Title/Abstract) or “thromboembolism” (Title/Abstract) or “embolization” (Title/Abstract) or “embolisation” (Title/Abstract) or “embolism” (Title/Abstract) or “emboli” (Title/Abstract).

#3 Search: “Budd-Chiari syndrome” (Title/Abstract) or “Budd-Chiari syndrome” (Mesh).

#4 Search: “factor V” (Mesh) or “factor V deficiency” (Mesh) or “factor Va” (Mesh) or “familial multiple coagulation factor deficiency I” (Supplementary Concept) or “factor V activating enzyme” (Supplementary Concept) or “factor V Leiden” (Supplementary Concept) or “factor V Leiden” (Title/Abstract) or “factor Va” (Title/Abstract).

#5 Search: “factor II” (Title/Abstract) or “prothrombin” (Title/Abstract) or “prothrombin” (Mesh).

#6 Search: #1 and #2.

#7 Search: #6 or #3.

#8 Search: #4 or #5.

#9 Search: #7 and #8.
